# The Relationship Between Dental Anxiety and Oral Health-Related Quality of Life in Patients with Periodontitis

**DOI:** 10.3390/diagnostics14232624

**Published:** 2024-11-22

**Authors:** Nicole Padilla-Fonseca, Agatha Araya-Castillo, María Paula Arias-Campos, Ana Paula Solís-Rivera, Jeniffer Jiménez-Matarrita, Karol Ramírez

**Affiliations:** 1 School of Statistics, University of Costa Rica, San Pedro, Montes de Oca, San José 11801, Costa Rica; nicole.padilla@ucr.ac.cr; 2 Faculty of Dentistry, University of Costa Rica, Finca 3 “Instalaciones Deportivas”, Sabanilla, Montes de Oca, San José 11502, Costa Rica; agatha.araya@ucr.ac.cr (A.A.-C.); maria.ariascampos@ucr.ac.cr (M.P.A.-C.); ana.solisrivera@ucr.ac.cr (A.P.S.-R.); jeniffer.jimenez@ucr.ac.cr (J.J.-M.)

**Keywords:** dental anxiety, periodontal disease, periodontitis, quality of life

## Abstract

Objectives: This study aimed to (1) investigate whether dental anxiety (DA) and oral health-related quality of life (OHRQoL) differed between patients diagnosed with periodontitis and individuals with periodontal health, (2) examine associations and correlations between these patient-reported measures, and (3) analyze demographic and clinical parameters. Methods: Ninety-six patients diagnosed with periodontitis and age- and sex-matched periodontally healthy controls were included. Participants’ demographic characteristics, smoking status, current dental pain, dental pain during the last month, the Modified Corah’s Scale (MDAS), and the Oral Health Impact Profile (OHIP-14) were determined. Results: The mean age of participants was 48.51 years ± 11.41. Patients with periodontitis experienced higher pain in the last month compared to controls (*p* = 0.003). Patients with periodontitis exhibited significantly higher MDAS total and sub-scores (*p* < 0.001). Compared to controls, the periodontitis group indicated extreme DA (1.04% vs. 7.79%, *p* = 0.034). Patients with periodontitis feared having a foreign object in the mouth compared to controls (*p* = 0.004). The periodontitis group exhibited worse OHIP-14 global and sub-scores (all *P*s < 0.001). Positive associations and correlations of MDAS total and sub-scores with OHIP-14 global and domain scores were found for the periodontitis group, but not for controls. Patients with periodontitis who reported “moderate and extreme anxiety” had poorer OHRQoL compared to controls *(p* = 0.001). The minimal importance difference for this finding indicates a large effect size and a moderate standardized response mean between groups. Conclusions: Patients with periodontitis had higher levels of DA and worse OHRQoL compared to controls. Our study highlights the importance of providing a comprehensive approach, including psychosocial well-being, when diagnosing and treating periodontal disease.

## 1. Introduction

Periodontitis is a chronic inflammatory disease associated with a dysbiotic biofilm in the subgingival environment of teeth [1]. In susceptible hosts, the exacerbated and dysregulated inflammatory response to bacterial products may result in the progressive destruction of tooth-support components [2]. In addition to clinical attachment loss, other characteristics of periodontal disease include gingival bleeding, gingival hyperplasia or gingival recession, presence of periodontal pockets, and radiographically assessed alveolar bone loss. If periodontal disease is left untreated, it may be the cause of halitosis, tooth mobility, and tooth loss. These clinical endpoints may adversely affect the quality of life of a person. Specifically, tooth loss may lead to masticatory dysfunction, speech alterations, and could negatively impact overall health, nutrition, and well-being [3,4]. Hence, patients with periodontitis should also be evaluated subjectively through patient reported outcomes (PROs) that quantify the impact of periodontal disease on quality of life [5].

Oral-health related quality of life (OHRQoL) reflects a person’s perception of how oral diseases and conditions affect different dimensions of a person’s well-being [6,7,8]. OHRQoL aids clinicians and public health policy makers to identify patient’s concerns, expectations and satisfaction of the provided therapy to promote oral health care and access to care [9]. The Oral Health Impact Profile short version (OHIP-14) is a validated instrument that measures OHRQoL in adults. The questionnaire includes fourteen functional and psychosocial impacts that measure functional limitations, discomfort, and disability. It has been translated into several languages and is accepted as a gold standard to evaluate OHRQoL.

Several studies using the OHIP-14 questionnaire have confirmed that periodontal disease impairs quality of life [5,8,10,11]. For example, a systematic review [12] indicated a significant influence of periodontal disease on the deterioration of OHIP-14 values, which was related to the degree of advancement of periodontitis and extent of periodontal tissue damage. Likewise, a systematic review and meta-analysis revealed that Stages III-IV periodontitis had a greater impact on OHRQoL than Stages I-II periodontitis [13]. A study comparing severity and rate of progression of periodontitis found that patients with Stage IV and Grade C periodontitis had the highest total OHIP-14 scores [14]. Studies from our group have shown significant improvements in OHRQoL after completion of the second step of periodontal therapy [15,16].

Research has evidenced that chronic and aggressive periodontitis is associated with worse OHRQoL and higher levels of anxiety to dental treatment [17,18]. Dental anxiety (DA) is defined as an excessive, disproportionate, and persistent nervousness or fear of dental procedures. Patients with DA avoid periodontal preventive measures, dental appointments, exhibit poor oral hygiene adherence or compliance, and maintain modifying lifestyle risk factors such as smoking, which culminates in deteriorating periodontal health and adversely affects OHRQoL [19]. Improvements in dental apprehension over the course of periodontal therapy have been analyzed [20] and associated with OHRQoL. Recently, our group correlated both OHRQoL and DA in individuals with periodontitis, before and after non-surgical periodontal treatment; as DA total and sub-scores increased, OHRQoL worsened [16].

To date, only a few studies have investigated the relationship between DA and OHRQoL in patients with periodontitis [17,18]. Understanding patient’s perceptions and needs is important for appropriate diagnosis and treatment planning. Therefore, the objective of this study was to examine whether DA and OHRQOL differed between people with periodontitis compared to age and sex-matched control individuals, diagnosed with periodontal health, based on the scheme of the 2017 World Workshop on the Classification of Periodontal and Peri-implant Diseases and Conditions. Additionally, we aimed to make correlations, associations, and partial correlation analyses between these two PROs. We further assessed the minimal important difference (MID) using the distribution-based approach to interpret clinically meaningful comparisons between groups’ OHRQoL and anxiety categories. We also analyzed demographic characteristics and clinical parameters among groups. We hypothesized that patients diagnosed with periodontal disease have higher DA levels and worse OHRQoL compared to patients diagnosed with periodontal health.

## 2. Materials and Methods

### 2.1. Study Design

This case–control observational study was conducted according to the Strengthening the Reporting of Observational Studies in Epidemiology (STROBE) guidelines [21]. This study was reviewed and approved by the Scientific Ethics Committee of the University of Costa Rica (CEC-517-2023) and conducted in accordance with the Helsinki Declaration of 1975, as revised in 2024.

### 2.2. Study Population

The present study was conducted between September 2023 and August 2024. Ninety-six patients with periodontal disease who attended the Clinic of Periodontics of the Faculty of Dentistry of the University of Costa Rica (FODUCR) were included. Only newly admitted patients with periodontitis were recruited. These patients never had subgingival instrumentation. Patients with a diagnosis of periodontitis based on the classification scheme of the 2017 World Workshop on the Classification of Periodontal and Peri-implant Diseases and Conditions [22] were recruited. All patients diagnosed with periodontitis received periodontal therapy after their participation in this study.

The control group consisted of ninety-six age and sex frequency-matched consecutive individuals diagnosed with periodontal health. These patients attended the Diagnostic Clinic of the FODUCR. All individuals in the control group had probing depths of ≤3 mm, in an intact or reduced periodontium, and had no known history of periodontal disease or treatment for periodontitis.

To be eligible to participate, patients had to be over 18 years old, diagnosed with periodontitis (cases) or with periodontal health (control group), with at least twenty teeth, with no cognitive impairments, and able to complete a questionnaire independently. Exclusion criteria included the use of illicit drugs, malignant diseases, taking medication for psychological disorders, sedatives, anxiolytics, or analgesics, pregnancy, or lactation, an acute dental or periodontal condition, and patients who had in the past had subgingival plaque instrumentation.

The sample size to ensure adequate power for this study was based on previous publications comparing OHRQoL of patients with and without periodontitis [17,18]. At least 192 patients, in two groups, with a 1:1 ratio of participants between the test group and the control group, were required to achieve 92.5% statistical power to identify a 4.48-point difference in the OHIP-14 global score, with an alpha set at 0.05 and an estimated standard deviation (SD) of 9.93 for the group with the highest SD and 8.25 for the group with the lowest SD.

### 2.3. Data Collection

The study was based on clinical examination and on a questionnaire. All patients underwent full periodontal charting (6 points per tooth) using a manual UNC-15 probe. A calibrated periodontist (KR) conducted periodontal probing examinations. The periodontal diagnoses were based on the 2017 World Workshop on the Classification of Periodontal and Peri-implant Diseases and Conditions (18). To define a patient as a periodontitis case, the individual had to present detectable interdental clinical attachment loss at ≥2 non-adjacent teeth, or buccal or oral clinical attachment loss of ≥3 mm with pocketing ≥ 3 mm, detectable at ≥2 teeth. Staging, or severity of periodontitis, was established as follows: Stage I (interdental clinical attachment loss of 1–2 mm or radiographic bone loss of <15%); Stage II (interdental clinical attachment loss of 3–4 mm or radiographic bone loss of 15–33%); Stage III (interdental clinical attachment loss of ≥5 mm or radiographic bone loss extending to the middle third of the root and beyond showed ≤ 4 teeth loss due to periodontitis); Stage IV (interdental clinical attachment loss of ≥5 mm or radiographic bone loss extending to the middle third of the root and beyond showed ≥ 5 teeth loss due to periodontitis). In addition, grading or rate of progression was determined as follows: Grade A, slow rate (no bone loss or attachment loss over five years or percentage of bone loss/age is <0.25, those affected are nonsmokers and those not diagnosed with diabetes); Grade B, moderate rate (<2 mm bone loss or attachment loss over 5 years or percentage of bone loss/age is 0.25–1.0, and risk factors are those who smoke < 10 cigarettes/day and have glycated hemoglobin (HbA1c) of <7.0%); Grade C, rapid rate (≥2 mm bone loss or attachment loss over 5 years or percentage of bone loss/age > 1, smoking +10 cigarettes/day, and HbA1c ≥ 7.0% in patients with diabetes). Patients defined as a periodontitis case were allocated in the periodontitis group.

Periodontal health or gingival health was determined for epidemiological purposes as described by Chapple et al. [23]. For an intact periodontium and a reduced and stable periodontium, gingival health was defined as <10% bleeding sites [24,25] with probing depths ≤ 3 mm. Patients defined as cases of gingival health on an intact and a reduced periodontium were allocated in the control group.

After clinical examination, a diagnosis was established, and a questionnaire was applied. The data collected included the following: (a) demographic characteristics: age, sex, and educational level attainment, (b) smoking status, and (c) dental pain. In addition, the Modified Corah’s Dental Anxiety Scale (MDAS), and (d) OHIP-14 were administered to assess DA and OHRQoL, respectively.

Participants were asked if they currently smoked. Smoking status was recorded as a yes or no response. We asked current smokers how many cigarettes they consumed per day.

Furthermore, the numeric rating scale (NRS) was used to record participants’ current intensity of dental pain and maximal pain during the last month. Each patient was asked to indicate on a scale of 0–10, where 0 represented “no pain”, 5 moderate pain, and 10 represented “worst pain imaginable”.

As mentioned, we used the MDAS Spanish version [26] to assess DA. This validated questionnaire consists of five questions. Each question describes a situation in the dental setting that might cause anxiety to treatment. The responses were scored. A score of 1 was rated as “not anxious” and a score of 5 was rated as “extremely anxious”. The responses to all the five questions were summed to determine each participant’s level of DA. The total sum could range from 5 to 25. Normal/slight anxiety was categorized as a score < 11, and scores between 11 and 18 represented moderate anxiety. Scores > 19 reflected extreme anxiety [27,28]. Additionally, we evaluated stimuli that may evoke DA. For this purpose, patients were asked to indicate a yes or no response, if the following phobic stimuli triggered anxious feelings: (a) dental injection; (b) the sound of rotatory instruments or the sound of the ultrasonic scaler noise; and (c) having a foreign object in the mouth [17,18].

As specified, we used the Spanish version of the OHIP-14 to determine OHRQoL. This validated questionnaire assesses seven domains: functional limitation, physical pain, psychological distress, physical disability, psychological disability, social disability, and disability. Each question has 5 response options. A score is assigned for each answer as follows: 0 = never; 1 = almost never; 2 = occasionally; 3 = frequently; 4 = always. A domain is made up of two questions and has a value that ranges from 0 to 8. The OHIP-14 total score is obtained by adding the 7 dimensions. Thus, scores vary between 0 and 56 [29]. Participants were asked the frequency of the impact in the last 6 months, for each of the questions that were asked.

### 2.4. Statistical Analysis

All statistical analysis were performed using R Studio software version 4.4.1. To test for normality, the Quantile Plot and the Shapiro–Wilk test were used. The null hypothesis was that the frequency distribution of the data was normally distributed. In this study, no variable met the assumption of normality.

Cronbach’s Alpha Coefficient was used to determine internal consistency of the MDAS and the OHIP-14 questionnaires. To analyze the hypothesis that there were differences between groups, the Wilcoxon rank test was used for quantitative variables. For binary variables, a sign test was used. Significance level was set at 5% (0.05). The Chi-square test was used to determine differences between MDAS categories. Cohen’s D test was used to measure effect size in OHRQoL. The effect size was calculated by dividing the standardized difference between means by the pooled standard deviations of the two groups [30].

Associations between MDAS total score and sub-scores with OHIP-14 total and domain scores for each group were determined using the Spearman Correlation and the Wilcoxon signed-rank test. Additionally, analysis of variance (ANOVA) was used to analyze differences among means. Also, Spearman’s partial correlation analysis between DA and OHRQoL was performed adjusting for age, gender, educational level, smoking status, and ex-smoker status. Statistical significance was set at *p* < 0.05.

Anxiety was characterized in two categories, which were participants with “normal /slight anxiety” and participants with “moderate and extreme anxiety”, to detect mean group differences between OHIP-14 global score and anxiety categories, using the Wilcoxon-signed rank test. The MID of OHIP-14 global score between DA categories were calculated using the distribution-based approach, for comparison between the periodontitis and the control group. The effect size (ES) and standardized response mean (SRM) were calculated. An ES and SRM of ≤0.2 indicates a small but clinically significant magnitude of difference, 0.3–0.7 a moderate difference, and 0.8 a large difference [30,31].

## 3. Results

The current study included one hundred and ninety-two adults: 96 participants diagnosed with periodontitis and 96 control individuals diagnosed with periodontal health, either on an intact periodontium or on a reduced periodontium. Patients in the control group attended the Diagnostic Clinic with the following treatment needs: 3% needed one or several dental extractions, 7% needed an endodontic treatment, 42% sought restorative dental care, and 48% needed a fixed/removable dental restoration.

Table 1 shows the demographic characteristics and smoking status of patients with periodontitis and the controls. Participants’ mean age was 48.51 ± 11.41 years (range 18–69 years). Significant differences were found between the periodontitis group and the control group in relation to attainment of secondary education (*p* = 0.021) and higher education (*p* < 0.001). Regarding smoking status, 10.42% of patients diagnosed with periodontitis were active smokers, whereas 7.29% of patients of the control group smoked. No significant differences were found for the prevalence of smoking between both groups, and in the number of cigarettes consumed per day (all *P*s > 0.05) (Table 1).

Table 1 also shows patients’ NRS pain scores. No differences were detected between current NRS scores in patients with periodontitis compared with the periodontally healthy controls. Nonetheless, the patients with periodontitis reported experiencing more pain during the last month compared to the periodontally healthy controls (*p* = 0.003).

The demographic characteristics of the patients with periodontitis by stage compared to the controls were analyzed and the results are presented in Table 2. The periodontitis group did not differ by stage when comparing age groups, sex, and smoking status. The study groups did not differ when comparing these variables. For clarity, age categorization was based on the definition of “adulthood” by the American Psychological Association [32], with a slight modification. Young adults were categorized as persons aged 18 to 35 years, middle-aged adults from 36 to 64 years, and older people aged 65 years and above. We modified the age range of young adulthood because most are legally identified as adults at 18 years of age in Costa Rica.

Table 3 presents demographic characteristics of the patients with periodontitis by grade compared to the controls. The periodontitis group did not differ by grade when comparing age groups, sex, and smoking status. The study groups did not differ when comparing these variables.

Cronbach’s alpha coefficient for the MDAS of patients with periodontitis was 0.875 and 0.723 for the control group, indicating good internal consistency. Table 4 presents phobic stimuli, MDAS categories, and MDAS total scores for the periodontitis and control groups. Compared with the controls, participants with periodontitis presented higher MDAS totals as well as sub-scores (*p* < 0.001). There were statistically significant differences in the distribution of MDAS categories between the periodontitis group and the control group. Compared with the controls, the periodontitis group exhibited a significantly higher percentage of patients with normal/slight anxiety (*p* = 0.033), moderate (*p* = 0.006), and extreme anxiety (*p* = 0.034). Compared to the controls, patients with periodontitis were more likely to fear having a foreign object in the mouth (*p* = 0.004). No significant differences were found between groups regarding fear of the sound of the ultrasonic scaler or fear of the sound of the rotatory instruments (*p* = 0.108). This question consists of two items referring to the sound or noise produced by dental instruments, which may trigger anxiety in the dental setting. Also, no differences were found between groups when asked if dental injections evoked anxious feelings (*p* = 0.104).

Cronbach’s alpha coefficient for the OHIP-14 in the periodontitis group was 0.901 and 0.773 in the control group, indicating a good level of internal consistency. The OHIP-14 global score and domain scores are presented in Table 5. Compared to the controls, the periodontitis group exhibited a higher OHIP-14 global score (*p* <  0.001; d = 0.84). Likewise, all domain scores were higher among the periodontitis group (all *P*s < 0.001). The highest scores for the periodontitis group were recorded on the physical discomfort domain (*p* <  0.001; d = 0.95), followed by psychological disability (*p* <  0.001; d = 0.96). The lowest scores were recorded on the social disability (*p* <  0.001; d = 0.58) and functional limitation (*p* <  0.001; d = 0.52) domains.

Table 6 shows correlations between MDAS total and sub-scores with OHIP-14 global and domain scores in the periodontitis group. A correlation coefficient of 0.1 to 0.3 indicates a weak or small linear relationship, 0.3 to 0.5 is considered a moderate linear relationship, 0.5 to 0.7 represents a strong correlation, and 0.7 to 1.0 indicates a very strong correlation [33]. A positive correlation was found between MDAS total with OHIP-14 global score (*r* = 0.567). This suggests a strong positive correlation between the OHIP global score and the MDAS total score. That is, as DA increases, the impact on OHRQoL also tends to increase. In the same line, MDAS sub-scores and OHIP-14 domain scores showed positive correlations (*r* = 0.092–0.517).

The ANOVA test confirmed that the mean of the MDAS total score was significantly associated with OHIP-14 global score in the periodontitis group (*p* < 0.001). Also, DA sub-scores were associated with a greater impact on all domains of OHRQoL, including functional limitation, physical pain, psychological distress, physical and psychological disabilities, social disability, and handicap (all *P*s < 0.05). In other words, a higher level of DA is related to a greater negative impact on OHRQoL.

Table 7 shows correlations and associations of MDAS total and sub-scores with OHIP-14 global and domain scores among the control group. MDAS total score and OHIP-14 global scores exhibit a weak positive correlation (*r* = 0.034). As for MDAS sub-scores and OHIP-14 domain scores, weak negative and positive correlations were assessed (*r* = −0.170–0.212). The ANOVA test confirmed that the mean of the MDAS total score was not significantly associated with OHIP-14 global score in the control group (*p* > 0.05). Also, DA sub-scores were not significantly associated with impact on domains of OHRQoL in the control group (all *P*s > 0.05).

Partial correlation analysis confirmed these results. After adjusting for age, the association between DA total score and OHIP total score in the patients with periodontitis was *r* = 0.564 and *r* = 0.060 in the control group. In the periodontitis group, as DA total score increases, OHIP-14 total score also tends to increase, assuming age remains constant. Similarly, after adjusting for gender, the association between the DA total score and OHIP total score in patients with periodontitis was *r* = 0.555 and *r* = 0.038 in the control group. After adjusting for educational level, DA total score and OHIP total score in the periodontitis group was *r* = 0.569 and *r* = 0.061 for the control group. And for smoking status and ex-smoking status, *r* = 0.565 in the patients with periodontitis and *r* = 0.059 in the control group.

Table 8 describes differences in OHRQoL (OHIP-14) between anxiety categories in the periodontitis and control group. DA was characterized in two categories which were “normal/slight anxiety” and “moderate and extreme anxiety”. Mean differences were significant for both the periodontitis and control group in the “moderate and extreme anxiety” category only. Thus, patients with periodontitis who reported “moderate to extreme anxiety” had poorer OHRQoL compared to the controls. The MID, according to the distribution-based approach, for the ES was 0.40 for the “normal/slight anxiety” category and 0.70 for the “moderate and extreme anxiety” category. The SRM was 0.26 for the “normal/slight anxiety” category and 0.54 for the “moderate and extreme anxiety” category. To clarify, an ES and SMR mean of ≤0.2 indicates a small but clinically significant difference between groups; 0.3–0.7 a moderate difference between groups, and ≥0.8 a large difference between groups.

## 4. Discussion

As far as we know, this is the first case–control study that evaluates, associates, and correlates both DA and OHRQoL in individuals with periodontal disease and periodontal health, using the OHIP-14 and MDAS questionnaires. We also evaluated self-assessment of pain and dental phobic stimuli. The recruited periodontal patients never had subgingival instrumentation and the control individuals did not have a history of periodontal disease or periodontal treatment. This study also addresses confounding factors such as demographic characteristics and smoking status.

The number of men and women who participated in this study did not differ significantly. The literature states that women tend to perceive a greater impact on their OHRQoL than men; therefore, women are more likely to visit their dentist for dental care compared to men [34,35]. In a gender-wise comparison among elderly patients with periodontitis, OHRQoL was found to be better among males, and no differences were found with different parameters of periodontitis [36]. Previous studies have reported that women experience more anxiety than men during dental treatment [37]. This has been explained by social roles, since women are more open to express their feelings, and it is more acceptable for women to express anxiety openly, whereas men are expected not to fear and show pain. However, other studies have not found differences in DA among sexes [38,39].

We determined education attainment since educational inequalities have been associated with an increased risk of periodontitis [40,41] and poor OHRQoL [28,42,43]. Previous investigations have revealed that individuals with higher educational level experience lower DA because they have a better understanding of treatment [44,45,46]. Furthermore, people with higher education levels are more aware of dental services and are more likely to attend dental appointments regularly. We found that patients with periodontitis had lower higher education attainment compared to controls. Similarly, two other studies found lower educational levels among patients with aggressive and chronic periodontitis compared to periodontally healthy controls [17,18]. It has been suggested that lower education results in less health literacy, and higher periodontal disease risk levels [47].

Smoking is a significant lifestyle/behavioral risk factor for periodontitis initiation and progression, which has profound effects on gingival tissues [48]. Smokers tend to report a lower oral health-related quality of life [49,50,51]. In terms of AD, active smokers are more likely to have dental fear compared to nonsmokers and those who use tobacco occasionally [52]. In the present study, no differences were detected for smoking status between the periodontitis and control group. Most of the participants were non-smokers. This may be due to Costa Rica’s General Law on Tobacco Control and its Harmful Effects on Health, No. 9028, which governs smoking in public spaces. Under this law, smoking or vaping is prohibited in shared spaces. Additionally, widespread anti-smoking campaigns in Costa Rica have led to a decline in the prevalence and consumption of tobacco cigarettes in recent years [53].

Generally, periodontal disease has no obvious pain component. As a result, symptoms such as bleeding gums are not the chief complaint of a patient when seeking dental treatment [54]. In the current study, no differences were found between groups on current pain. Nonetheless, patients with periodontitis reported feeling more pain during the last month than individuals in the control group. The literature states that only 6.2% of people with periodontitis report having painful gingiva [55]. This may be related to a severe stage of the disease [56] where attachment loss, clinical mobility, and radiographic bone loss can be detected. Loss of attachment due to periodontal diseases is associated with functional limitations as well as physical pain and psychological discomfort, which may negatively impact the OHRQoL [57]. It should be noted that most of the participants in the periodontitis group were diagnosed with Stage III-IV and Grade B-C.

DA manifests as emotional fear triggered by dental stimuli. Patients cite fear-inducing stimuli in the dental setting, such as injections, the sound of drills or ultrasonic devices, and the pain associated with dental procedures [58]. DA may result in postponing periodontal therapy and maintenance. Levin et al. observed that patients with periodontitis more likely feared the noise of dental instruments, the application of dental injections, and the idea of having a foreign object in the mouth [17]. Likewise, patients with aggressive periodontitis were more likely to fear the dental drill noise and having a foreign object in the mouth, compared to participants with periodontal health [18]. In our study, we found no differences between groups when asked about fearing dental injections and if the sound of rotatory instruments or ultrasonic scaler noise evoked fear. However, patients with periodontitis were more likely to fear having a foreign object in their mouth, compared to controls. None of the participants diagnosed with periodontitis had in the past any form of periodontal treatment for gum disease. It has been proven that over 70% of patients referred for periodontal treatment who had not previously undergone any periodontal therapy felt anxious about their upcoming treatment [59]. Thus, we assume that our patients who were diagnosed with periodontitis may have feared or were distressed about an uncertain situation such as a new method of dental treatment. In a recent study from our group, we found that patients experienced less fear of having a foreign object in their mouth, after Steps 1 and 2 of periodontal therapy [16,20].

In the current study, the periodontitis group exhibited significantly higher scores in the MDAS total and sub-scores, compared with their corresponding control group. The mean MDAS total score of the periodontitis group was 10.92 ± 4.75 points. This score is like the one reported by Tu et al., 10.53 ± 4.07 points, who compared two patient groups diagnosed with periodontitis before and after non-surgical periodontal therapy. As hypothesized, the control group in our study reported experiencing normal/slight anxiety to dental treatment. Patients are generally anxious about any type of dental treatment. Therefore, the dental professional should consider a comprehensive approach to help patients cope with DA. In the current work, moderate and extreme anxiety was more prevalent among patients with periodontitis compared to controls. The presence of deep dental pockets, greater loss of clinical attachment level, and complex periodontal needs have been associated with moderate-to-high DA [60,61].

In the present investigation, patients with periodontitis exhibited worse OHIP-14 global as well as worse domain scores, compared to controls. This is in line with an umbrella review of systematic reviews which concluded that periodontal disease is negatively correlated with OHRQoL, and treatment can improve self-reported quality of life [62]. One systematic review concluded that physical (functional) and psychological domains were the two most associated ones with periodontitis [63]. Functional limitation relates to loss of teeth functionality, worsened sense of taste, trouble in pronouncing words, and severe tooth loss. Mobility and tooth loss as an endpoint of periodontitis might compromise function. Functional limitations may lead to poor chewing and consequently digestive discomfort if the patient does not recur to prosthetic rehabilitation [64]. Attachment loss due to periodontal diseases has been related to functional limitations as well as physical and psychological discomfort [58].

Effect size describes the magnitude of the difference between groups [65]. In the current study, the effect size in the OHRQoL global scores between groups was large, *d* = 0.84. We found a large effect or magnitude of difference between groups in the domains of psychological discomfort, psychological disability, and handicap. Psychological discomfort is manifested as being unhappy with aesthetics, specifically the appearance of teeth. Psychological disability is described as “having to interrupt meals” and describing “having an unsatisfactory diet”. Research studies have evidenced that the scores for these domains bettered significantly after periodontal treatment [66,67,68,69], suggesting that the most important periodontal-related problems perceived by patients diagnosed with periodontitis are embarrassment, stress, and difficulty relaxing [12]. Periodontitis may affect social interactions and enjoying daily life activities.

A positive correlation and association were found between MDAS total score and sub-scores with OHIP-14 global and domain scores, in patients with periodontitis, but not for the control group. An evaluation of DA using validated questionnaires in routine clinical practice for the proper management of anxiety to dental treatment could consequently improve a patient’s experience of dental care and OHRQoL. Based on anxiety questionnaires, patients can be classified as experiencing low-, moderate-, or high-anxiety levels. Management of DA should be tailored specifically to patients’ level of anxiety, age, gender, educational level, and clinical situation [70]. Pharmacotherapeutic interventions are available to ameliorate high levels of DA, these include sedation or general anesthesia. Also, psychotherapeutic management or a combination with pharmacotherapy are recommended. Exposure therapy, which involves a systematic encounter with stimuli, such as objects and situations that evoke distress, has been proposed in the management of DA.

Anxiety levels were further characterized in two categories, which were “normal/slight anxiety” and “moderate and extreme anxiety” to verify differences in OHRQoL between groups, using the OHIP-14 total score as a reference. We found no significant differences between groups’ OHIP-14 total score for individuals experiencing “normal/slight anxiety” to dental treatment. Nonetheless, we found significant differences between groups in the “moderate and extreme anxiety” category. According to our results, patients with periodontitis who reported “moderate and extreme anxiety” had poorer OHRQoL compared to controls. The MID, using the distribution-based approach, indicates the ES among groups is large and has a moderate SRM difference. This is in line with other reports in other geographical areas, with other oral diseases, that have found a relevant association between increased DA and OHRQoL, a greater DA score being associated with lower OHRQoL [71,72,73]. One of the limitations of our study was that we did not use an anchor-based approach, which would have provided more evidence that the differences observed between groups were meaningful [31]. A recommendation is to execute a follow-up study that considers patients’ responses to global transition questions, for the self-assessment of oral health status as a reference criterion. As suggested in a previous report, global questions asking how individuals rate their oral health could be (1) excellent; (2) very good; (3) good; (4) fair; and (5) poor [74].

This study showed the negative impact of DA on OHRQoL, particularly in patients with periodontitis. A systematic review identified a correlation between clinical attachment loss, periodontal pocket depth, and periodontitis severity with higher anxiety scores [75]. Reports have specified recommendations for dental practitioners on how to integrate DA management strategies into periodontal care, particularly for patients who exhibit moderate to severe anxiety [20,76]. Essentially, good communication between the patient and the dentist is crucial to manage DA. There is a need to emphasize the empathy of the clinician, to gain the patient’s trust [76]. The stepwise approach of periodontal therapy may be split into several sessions instead of a full mouth treatment approach. To illustrate, the first step of periodontitis treatment is the phase aimed at guiding and motivating patients to change behavioral practices to undertake successful removal of supragingival dental biofilm and control risk factors associated with periodontitis [77]. The first dental appointment of a patient with high anxiety may be limited to the first step of periodontal care. The second step, or cause-related therapy, may be performed in several sessions. Subgingival instrumentation may be performed in four different sessions, by sections or quadrants. With time, patients may realize that the situations in periodontal therapy cause low levels of pain and discomfort, thus reducing levels of DA [20].

Despite careful screening to exclude patients taking medication for psychological disorders, such as sedatives and anxiolytics that could impact anxiety assessment, patients’ answers may generate significant variability according to the momentary perception of each participant and reasons to seek dental care. Also, oral health complaints and awareness may affect patients’ responses. It is possible that other oral conditions present in patients with periodontal disease may have influenced greater DA and lower OHRQoL scores. Additionally, there is currently a lack of suitable instruments to specifically assess anxiety to periodontal therapy. Also, specialized Oral Health Impact Profile questionnaires have been designed to evaluate how the most common symptoms of periodontal disease impact well-being. Consequently, a limitation of the current study is that some of the questions in OHIP-14 are not pointedly related to periodontal diseases. Another limitation to consider is that this study reflects the PROs of patients attending a university-based dental clinic and not necessarily that of the community.

This study relied on self-reported data, which may introduce bias. Self-report bias occurs when participants in a study are asked to describe their feelings, thoughts, or behaviors. There might be a deviation between the self-reported information and true values of a measurement. This bias may occur particularly in the assessment of anxiety levels, which could be influenced by the social desirability of responses. For instance, participants who exhibit moderate to high anxiety may give more socially acceptable answers. In this line, women are more prone to express their feelings about DA and phobia. Conversely, men tend to hide their emotions due to cultural and social stereotypes [78]. Also, individuals may not properly be able to accuretely evalute their anxiety levels on a Likert scale and may give extreme or middle responses to all questions.

The strengths of this study include an appropriate sample size of patients diagnosed with periodontitis and periodontally healthy controls who had never received any kind of periodontal treatment. We used standardized and internationally validated questionnaires, the MDAS and OHIP-14, and the visual analog scale to determine current dental pain and dental pain during the last month. Another strength and novelty is that we assessed diagnosis of periodontitis and periodontal health using the current classification of periodontal disease.

## 5. Conclusions

In the present study, DA and OHRQoL differed between patients with periodontitis compared to age and sex-matched control individuals. DA and OHRQoL were correlated and associated in patients with periodontitis only; as DA total and sub-scores increased, OHRQOL worsened. DA and OHRQoL are both PROs of pivotal importance in dental care. DA may be a barrier in the access to periodontal treatment, while OHRQoL is of central significance when trying to understand the impact of periodontitis and the effectiveness of interventions on a patient’s well-being. Periodontitis has negative impacts on functional, psychological, and social dimensions, compared to matched periodontally healthy patients. Clinicians should take into consideration these impacts when diagnosing and treating patients with periodontal disease to satisfy patients’ expectations and needs.

Particular attention should be paid to patients who avoid dental visits and focus on educating and informing them about dental procedures, to reduce DA. There are several validated short questionnaires to screen DA, such as the MDAS. Clinicians could rely on a regular basis on these tools since it is not easy to identify fearful patients in dental practice. It is important to consider the patient’s level of DA to provide a comprehensive care plan. Sedation and general anesthesia are options when treating patients with high levels of DA and phobia. Nonetheless, this is a temporary solution and does not help individuals to overcome their fears. Interdisciplinary management by psychologists and dentists is recommended. Psychologists could prepare a patient to receive dental treatment, by explaining every step of the therapy and gradually introducing the dental setting environment. Clinicians could consider treatment staging and longer appointments to help patients cope progressively with their fears. Developing strategies to identify patients with severe anxiety may improve their experience in the dental office and adherence to treatment and overall treatment outcomes, including well-being.

There may be potential barriers when implementing anxiety-reduction strategies in clinical practice, such as time constraints, cost, and the need for additional training. For instance, in dental emergencies, there may not be enough time to implement psychotherapeutic interventions or a gradual stepwise treatment plan. Thus, in these cases, pharmacological interventions would be the best option to treat patients with high levels of DA. Another barrier is the cost of being attended to by an interdisciplinary team. Therefore, dentists should consider additional training in DA management strategies such as relaxation techniques, cognitive behavioral therapy, and sedation.

## Figures and Tables

**Table 1:** Demographic characteristics, smoking status, current pain scores, and pain during the last month scores of patients with periodontitis compared to controls.

Parameter	Variable	Periodontitis Group	Control Group	*p* Value *
		No. (%)	No. (%)	
Sex	Male	39 (40.62)	38 (39.59)	1.000
	Female	57 (59.38)	58 (60.41)	
				*p* value *
Education level	Primary	24 (25.00)	13 (13.54)	0.067
	Secondary	39 (40.63)	23 (23.96)	**0.021**
	Higher education	27 (28.12)	56 (58.33)	**<0.001**
	None-degree program	6 (6.25)	4 (4.17)	0.745
Smoking status	Yes	10 (10.42)	7 (7.29)	0.629
	No	86 (89.58)	89 (92.71)	
		Mean	SD	*p* value **
Number of cigarettes per day	Periodontitis	5.90	5.88	0.214
	Control group	2.57	1.40	
Age		48.51	11.41	
Current pain	Periodontitis	1.44	2.49	0.630
	Control group	1.29	2.66	
Maximal pain in the last month	Periodontitis	3.13	3.31	**0.003**
	Control group	1.82	2.87	

* Chi-squared test, ** Wilcoxon test, *p* values in bold denote statistical significance (*p* < 0.05), SD, standard deviation.

**Table 2:** Demographic characteristics of patients with periodontitis by stage compared to controls.

Demographic Characteristics	Stage I No (%) *n* = 2	Stage II No (%) *n* = 23	Stage III No (%) *n* = 60	Stage IV No(%) *n* = 11	*p* Value *	Control Group
Age (years)					0.354	
18–35 (young)	1	6	8	0		14
36–64 (middle-aged)	1	16	50	10		77
65 (older adults)	0	1	2	1		5
Sex					0.576	
Male	0	8	26	5		38
Female	2	15	34	6		58
Smoking					0.199	
Yes	0	3	4	3		7
No	2	20	56	8		89

* Chi-squared test.

**Table 3:** Demographic characteristics of patients with periodontitis by grade compared to controls.

Demographic Characteristics	Grade A. No (%) *n* = 9	Grade B. No (%) *n* = 45	Grade C. No (%) *n* = 42	*p* Value *	Control Group
Age (years)				0.788	
18–35 (young)	1	9	5		14
36–64 (middle-aged)	8	34	35		77
65 (older adults)	0	2	2		5
Sex				0.864	
Male	3	18	18		38
Female	6	27	24		58
Smoking					
Yes	0	4	6	0.400	7
No	9	41	36		89

* Chi-squared test.

**Table 4:** Phobic stimuli, Modified Dental Anxiety Scale (MDAS) categories, and MDAS total scores, as indicated by patients with periodontitis and controls.

**Phobic Stimuli**	**Variable**	**Periodontitis**		**Control Group**		*p* Value *
		**No. (%)**		**No. (%)**		
Dental injections	Yes	47 (48.96)		35 (36.46)		0.104
	No	49 (51.04)		61 (63.54)		
Sound of rotatory	Yes	33 (34.38)		22 (22.92)		0.108
instruments or ultrasonic scaler noise	No	63 (65.63)		74 (77.08)		
Foreign object in the mouth	Yes	25 (26.04)		10 (10.42)		**0.004**
	No	71 (73.96)		86 (89.58)		
						*p* value **
MDAS categories	Normal/slight	56 (58.33)		81 (84.38)		**0.033**
	Moderate anxiety	33 (34.38)		14 (14.58)		**0.006**
	Extreme anxiety	7 (7.29)		1 (1.04)		**0.034**
**Parameter**		**Periodontitis**		**Control Group**		*p* Value *
		**Mean**	**SD**	**Mean**	**SD**	
Visit tomorrow		2.04	1.15	1.42	0.84	**<0.001**
Waiting room		1.89	0.99	1.38	0.81	**<0.001**
Use of drill		2.26	1.22	1.39	0.70	**<0.001**
Scale and polish		2.23	1.16	1.30	0.56	**<0.001**
Injection		2.50	1.27	1.85	1.02	**<0.001**
Total MDAS score		10.92	4.75	7.33	2.76	

* Sign test ** Chi-squared test, *p* values in bold denote statistical significance (*p* < 0.05), SD, standard deviation.

**Table 5:** Mean Oral Health Impact Profile (OHIP-14) global and domain scores of patients with periodontitis compared to controls.

OHIP-14		Mean	SD	Effect Size *	*p* Value **
OHIP-14 global score	Periodontitis	15.97	15.00	0.84	**<0.001**
	Control Group	6.85	10.46		
Functional Limitation (OHIP-1 + 2)	Periodontitis	1.01	1.63	0.52	**<0.001**
	Control Group	0.32	0.92		
Physical pain (OHIP-3 + 4)	Periodontitis	2.59	2.22	0.58	**<0.001**
	Control Group	1.41	1.76		
Psychological discomfort (OHIP-5 + 6)	Periodontitis	4.52	2.50	0.95	**<0.001**
	Control Group	2.27	2.22		
Physical disability (OHIP-7 + 8)	Periodontitis	1.91	2.25	0.40	**<0.001**
	Control Group	1.06	1.99		
Psychological disability (OHIP-9 + 10)	Periodontitis	3.05	2.35	0.96	**<0.001**
	Control Group	1.10	1.67		
Social disability (OHIP-11 + 12)	Periodontitis	0.97	1.66	0.58	**<0.001**
	Control Group	0.23	0.67		
Handicap (OHIP-13 + 14)	Periodontitis	1.92	2.39	0.77	**<0.001**
	Control Group	0.46	1.23		

* An effect size of ≤0.2 indicates a small but clinically significant difference, 0.3–0.7 a moderate difference, and >0.7 a large difference, ** Wilcoxon test, *p* values in bold denote statistical significance (*p* < 0.05), SD, standard deviation, OHIP, Oral Health Impact Profile.

**Table 6:** Correlations and associations of MDAS total and sub-scores with OHIP-14 global and domain scores among patients with periodontitis.

	Functional Limitation (OHIP-1 + 2)	Physical Pain (OHIP-3 + 4)	Psychological Discomfort (OHIP-5 + 6)	Physical Disability (OHIP-7 + 8)	Psychological Disability (OHIP-9 + 10)	Social Disability (OHIP-11 + 12)	Handicap (OHIP-13 + 14)	OHIP-14 Global Score
Total MDAS score *	0.225	0.402	0.512	0.417	0.510	0.360	0.459	0.567
**	**<0.001**	**<0.001**	**<0.001**	**<0.001**	**<0.001**	**<0.001**	**<0.001**	**<0.001**
Visit tomorrow *	0.092	0.293	0.450	0.281	0.405	0.247	0.440	0.457
**	**0.024**	**0.002**	**<0.001**	**0.004**	**<0.001**	**0.011**	**<0.001**	**<0.001**
Waiting room *	0.157	0.371	0.427	0.398	0.455	0.325	0.380	0.498
**	**0.001**	**<0.001**	**<0.001**	**<0.001**	**<0.001**	**<0.001**	**<0.001**	**<0.001**
Use of drill *	0.235	0.328	0.400	0.386	0.385	0.261	0.335	0.449
**	**0.001**	**0.002**	**<0.001**	**<0.001**	**<0.001**	**0.015**	**<0.001**	**<0.001**
Scale and polish *	0.303	0.378	0.515	0.371	0.437	0.292	0.409	0.517
**	**<0.001**	**<0.001**	**<0.001**	**<0.001**	**<0.001**	**0.002**	**<0.001**	**<0.001**
Injection *	0.249	0.260	0.270	0.279	0.373	0.321	0.290	0.379
**	**0.002**	**0.003**	**0.009**	**0.015**	**<0.001**	**<0.001**	**0.011**	**<0.001**

* Spearman correlation, ** ANOVA means’ association, *p* values in bold denote statistical significance (*p* < 0.05).

**Table 7:** Correlations and associations of MDAS total and sub-scores with OHIP-14 global and domain scores among the control group.

	Functional limitation (OHIP-1 + 2)	Physical Pain (OHIP-3 + 4)	Psychological Discomfort (OHIP-5 + 6)	Physical Disability (OHIP-7 + 8)	Psychological Disability (OHIP-9 + 10)	Social Disability (OHIP-11 + 12)	Handicap (OHIP-13 + 14)	OHIP-14 Global Score
Total MDAS score *	−0.014	−0.063	0.191	−0.118	−0.025	0.094	−0.132	0.034
**	0.514	0.283	0.053	0.335	0.848	0.453	0.596	0.570
Visit tomorrow *	−0.150	0.085	0.092	0.016	0.002	0.183	−0.053	0.091
**	0.241	0.132	0.168	0.928	0.352	0.384	0.948	0.294
Waiting room *	0.004	−0.035	0.176	−0.116	−0.019	0.150	−0.051	0.076
**	0.959	0.338	0.107	0.200	0.200	0.483	0.878	0.544
Use of drill *	−0.170	−0.050	0.108	−0.059	−0.107	0.105	−0.142	−0.053
**	0.210	0.998	0.294	0.545	0.494	0.328	0.347	0.801
Scale and polish *	−0.011	−0.077	−0.021	0.043	−0.137	0.032	−0.033	−0.096
**	0.790	0.936	0.617	0.773	0.447	0.367	0.966	0.930
Injection *	0.074	−0.017	0.212	−0.152	0.010	−0.040	−0.155	0.046
**	0.869	0.340	0.070	0.155	0.883	0.677	0.484	0.753

* Spearman correlation, ** ANOVA means’ association.

**Table 8:** Differences in OHRQoL (OHIP-14) between anxiety categories in patients with periodontitis and control group.

	Normal/Slight Anxiety	Moderate and Extreme Anxiety
OHIP-14		
*N* with periodontitis	36	60
Mean score patients with periodontitis (SD)	5.19 (3.66)	22.43 (9.45)
*N* control group	73	23
Mean score patients without periodontitis (SD)	3.86 (3.18)	16.35 (5.97)
Difference	1.33	6.08
*p* value * (within group)	0.057	**0.001**
MID		
Distribution-based approach		
ES **	0.40	0.70
SMR **	0.26	0.54

*N*, sample size, * Wilcoxon test, *p* values in bold denote statistical significance (*p* < 0.05), MID, minimal important difference, ** An effect size (ES) and standardized response mean (SMR) of ≤0.2 indicates a small but clinically significant difference; 0.3–0.7 a moderate difference, and ≥0.8 a large difference.

## Data Availability

The datasets used and/or analyzed during the current study are available from the corresponding author on reasonable request.
